# Multimodal AI fusion: integrating MRI with PET/CT, histopathology, and liquid biopsy for bone tumor diagnosis

**DOI:** 10.3389/fonc.2026.1724332

**Published:** 2026-07-27

**Authors:** Guochao Li, Yayun Lin, Xueling Wang

**Affiliations:** 1 Department of Imaging, Yantaishan Hospital, Yantai, Shangdong, China; 2 Department of Ultrasonography, Yantaishan Hospital, Yantai, Shangdong, China

**Keywords:** AI−assisted decision support, bone tumor diagnosis, data integration in oncology, digital tumor board, multimodal artificial intelligence, precision oncology, precision oncology workflow

## Abstract

The diagnosis of bone tumors, though a low-incidence occurrence, presents a formidable challenge due to the morphological heterogeneity and variable biological behavior of these lesions. No single diagnostic modality whether imaging or biopsy is sufficient to provide the comprehensive information necessary for definitive diagnosis, accurate staging, and effective treatment planning. This report argues that a fragmented, sequential approach to diagnosis is inherently limited and that the future of orthopedic oncology lies in the integration of diverse data streams. Multimodal artificial intelligence (AI) offers a promising supportive approach by creating an AI−enabled integrative decision-support environment where computational systems can synthesize and contextualize information from magnetic resonance imaging (MRI), positron emission tomography computed tomography (PET/CT), histopathology, and liquid biopsy. This report details the specific capabilities and limitations of each of these foundational modalities, outlines the technical architectures required for their fusion, and illustrates how an integrated AI system can augment clinical workflows. It also addresses the critical challenges that must be overcome for clinical adoption, including data standardization, algorithmic interpretability, and economic viability, ultimately positioning this approach as an important emerging direction for future precision oncology.

## Introduction

The realm of bone tumor diagnosis is undergoing a paradigm shift, propelled by the fusion of diverse diagnostic modalities-magnetic resonance imaging (MRI), positron emission tomography/computed tomography (PET/CT), histopathology, and liquid biopsy-through advanced artificial intelligence (AI) methodologies. This multimodal integration aims to support the physician’s expert synthesis of diverse data points, addressing the significant cognitive load presented by the increasing complexity of high-dimensional data ranging from high-resolution MRI to genomic liquid biopsies. Rather than focusing on the inherent limitations of individual modalities, this approach provides a quantitative decision-support framework that assists clinicians in systematically cross-referencing findings that might otherwise remain siloed, thereby ensuring a more holistic and nuanced picture of bone tumor biology (1–5).

AI-driven multimodal fusion has demonstrated dramatic progress in recent years, buttressed by advancements in deep learning architectures, high-throughput bioinformatics, data harmonization, and clinical trial validation. Importantly, the clinical deployment of these integrative frameworks faces persistent challenges related to data heterogeneity, model interpretability, workflow integration, and regulatory scrutiny (4, 6). Crucially, the practical necessity for this integration is rooted in the complexity of real-world clinical decision-making. In orthopedic oncology, clinicians frequently encounter ‘diagnostic discordance’ scenarios where anatomical features on MRI may be inconclusive, while metabolic activity on PET/CT suggests potential malignancy. Multimodal AI serves as a vital decision-support tool in these ambiguous cases, providing a synthesized probability index that directly informs critical choices such as determining surgical margins and the optimal timing for neoadjuvant chemotherapy. By moving beyond subjective, sequential interpretations, this integrated approach enhances the objectivity of multidisciplinary tumor boards. As such, this comprehensive narrative review synthesizes contemporary advances in AI-powered fusion of MRI, PET/CT, histopathology, and liquid biopsy modalities. It highlights recent breakthroughs, deep learning architectures, preprocessing and harmonization strategies, validation evidence, interpretability solutions, and the regulatory and translational landscape. By mapping the present and future of multimodal AI in bone tumor diagnosis, this review aims to provide a definitive resource for clinicians, computer scientists, regulators, and industry stakeholders.

## The diagnostic imperative: a framework for advancing bone tumor classification

Primary malignant bone tumors (PBTs) are rare and morphologically diverse, often leading to diagnostic delays and variability among non-specialists (7). The traditional sequential, manual integration of disparate modalities relying on disconnected studies like radiographs and MRI fails to systematically integrate heterogeneous data, creating a diagnostic gap prone to cognitive biases (8, 9). This necessitates a reliance on more advanced and cross-sectional modalities like MRI, CT, and PET/CT, which provide complementary information to characterize the lesion and assess its aggressive nature (10). This reliance on a series of disconnected, unimodal studies highlights a fundamental diagnostic gap: the inability of the human diagnostic process to consistently and systematically synthesize a vast array of heterogeneous data points. The traditional clinical workflow depends on a physician’s ability to mentally integrate information from imaging, patient history, lab findings, and biopsy results. This process, however, is prone to cognitive biases and processing limitations, which are the root causes of diagnostic discrepancies and inconsistencies (8).

In this context, artificial intelligence (AI) has emerged not as a replacement for human expertise but as a crucial augmentative tool to revolutionize medical diagnosis. AI-driven systems leverage extensive databases and complex algorithms to identify subtle patterns and irregularities that can easily escape human perception. The value of AI lies in its ability to enhance diagnostic accuracy, enable earlier detection of disease, and significantly improve workflow efficiency by automating time-consuming analyses (11). AI-based models have demonstrated performance metrics that are on par with or, in some instances, even superior to non-specialist clinicians, with the potential to reduce misdiagnosis rates among doctors who are not musculoskeletal oncology specialists. The unique strength of multimodal AI is its capacity to systematically fuse disparate data types into a unified analytical framework. This approach can detect intricate patterns and relationships that exist across different data sources, effectively creating a more comprehensive and objective “diagnostic portrait” of the patient’s condition (12). By directly addressing the root causes of diagnostic subjectivity and inconsistency, multimodal AI presents a promising supportive approach to the long-standing challenges in bone tumor classification.

## The multimodal AI paradigm: principles and architectural foundations

Multimodal AI represents a groundbreaking approach in healthcare that addresses the limitations of traditional, single-data-stream analyses. It involves the integration of heterogeneous data sources such as medical images, structured electronic health records (EHRs), physiological signals, and genomic information into a unified analytical framework (13–16). Data fusion is the core computational process that enables this integration, combining data from different modalities to provide a more comprehensive view of a patient’s health and reduce diagnostic errors. The methods for achieving this fusion are critical to the design and clinical utility of the AI model.

The primary architectural approaches to data fusion are categorized into three main strategies, each with distinct trade-offs. Early Fusion: This method combines raw data from different modalities at the input stage, encoding them into a single, shared embedding space (17). An example of this is the fusion of MRI and PET images into a single composite image, such as a “GM-PET” image, which combines anatomical and metabolic information into one modality for subsequent analysis. The primary advantage of this approach is its capacity for a strong contextual understanding between modalities, which can lead to improved retrieval accuracy because the model can identify a tight coupling between data types. However, early fusion is computationally expensive, requires large, perfectly aligned multimodal datasets for training, and lacks flexibility, as adding a new data modality often necessitates retraining the entire system (17). Late Fusion: In contrast, late fusion processes each modality independently using a separate model and then combines the outputs later in the pipeline (18). An illustrative example is a voting ensemble model that combines the malignancy probabilities from separate image models trained on different MRI sequences (T1W and T2W) with the probabilities from a logistic regression model trained on clinical data (age, sex, and lesion location) (19). This modularity is a significant strength, as it simplifies updates and allows for the use of specialized, optimized models for each data type. The primary drawback of this approach is the risk of missing subtle but important cross-modal relationships that are not evident when modalities are processed in isolation. Hybrid Fusion: This strategy seeks to balance the trade-offs of early and late fusion by combining elements of both (20). These approaches often employ modality-specific encoders with a shared indexing layer. For instance, the Primary Bone Tumor Classification Transformer Network (PBTC-TransNet) is a hybrid model designed to classify bone tumors from incomplete multimodal images (X-ray, CT, MRI) and clinical characteristics, effectively mirroring real-life clinical scenarios where complete data is often unavailable (21, 22). This architectural choice highlights a crucial understanding: for a rare and heterogeneous disease like bone cancer, obtaining a large, perfectly aligned dataset for early fusion is incredibly difficult and logistically impractical. This makes late and hybrid fusion strategies, which are designed to operate effectively with incomplete data, particularly valuable for real-world clinical deployment. Their robustness and ability to function in data-scarce environments are often more important for clinical utility than a marginal increase in peak performance under idealized conditions (21).

Central to these fusion strategies are foundational deep learning architectures. Convolutional Neural Networks (CNNs) are the workhorse for analyzing image data, excelling at spatial pattern recognition for tasks like tumor detection, segmentation, and classification. Recurrent Neural Networks (RNNs) are better suited for sequential data, such as longitudinal patient histories or genomic sequences (23). More recently, advanced models like Transformers and attention mechanisms have shown great promise in capturing complex interactions across diverse data types, proving valuable for unifying information from clinical notes, imaging, and genomics into a single framework (24) (**Table 1**).

**Table 1:** Data fusion architectures in multimodal AI for bone tumor diagnosis.

Fusion strategy	Definition	Key advantages	Limitations	Clinical example in bone oncology	References
Early Fusion	Combines raw data from different modalities at the input stage into a single shared embedding space.	Strong contextual understanding; captures tight coupling between data types (e.g., anatomical + metabolic).	Computationally expensive; requires large, perfectly aligned datasets; lacks flexibility for missing data.	Fusion of MRI and PET images into a single “GM-PET” composite image for joint analysis	(17).
Late Fusion	Processes each modality independently using separate models and combines the final outputs (e.g., probabilities).	Modular and flexible; allows use of specialized models for each data type; robust to missing modalities.	May miss subtle cross-modal relationships that are not evident when processed in isolation.	Voting ensemble combining malignancy probabilities from separate MRI models and clinical logistic regression models	(19).
Hybrid Fusion	Combines elements of both early and late fusion, often using modality-specific encoders with a shared indexing layer.	Balances performance and flexibility; effective in real-world scenarios with incomplete or heterogeneous data.	Complex architecture design; requires careful tuning of integration layers.	PBTC-TransNet: Classifies tumors using X-ray, CT, MRI, and clinical data, even when some modalities are missing	(21, 22).

## Core diagnostic modalities in orthopedic oncology: strengths and AI-enhanced roles

The traditional diagnostic workup for a suspected bone tumor is a multi-step process that utilizes a range of imaging modalities and histopathological examination, each providing a unique piece of the puzzle. The true power of a multimodal approach is revealed when these data sources are viewed not in isolation, but as complementary layers of information that an integrated AI system can systematically analyze to enable a comprehensive diagnosis.

Despite the sophistication of advanced cross-sectional imaging, plain radiographs remain the indispensable first-line modality in the diagnostic algorithm for bone tumors. They provide immediate critical information regarding lesion morphology, matrix mineralization, periosteal reactions (e.g., Codman triangle, sunburst pattern), and cortical integrity, which are essential for initial triage (7, 10). Classic benign entities, such as non-ossifying fibromas, can often be diagnosed confidently on plain films alone, allowing for a “leave-alone” approach without further invasive workup. Conversely, aggressive features like cortical breakthrough guide the urgency for advanced staging. Recently, artificial intelligence has significantly revitalized the utility of plain radiographs. Deep learning models, particularly Convolutional Neural Networks (CNNs), have demonstrated robust performance in classifying bone tumors directly from X-rays, often matching the diagnostic accuracy of musculoskeletal radiologists (9, 37). Radiomic analyses of plain films have also shown promise in predicting tumor histology and grade, providing a low-cost, accessible tool for initial risk stratification (42). Integrating AI-analyzed radiographs into multimodal frameworks ensures that this foundational diagnostic data is fully leveraged alongside advanced modalities like MRI and PET/CT.

While plain radiographs serve as the initial screening tool, Computed Tomography (CT) remains indispensable for detailed characterization of bone tumors. CT provides superior spatial resolution for assessing cortical integrity, detecting subtle periosteal reactions, and characterizing tumor matrix mineralization (e.g., distinguishing osteoid from chondroid matrices), which are often difficult to discern on plain films or MRI (10, 25). Furthermore, CT is the gold standard for staging primary bone sarcomas, particularly for the detection of pulmonary metastases, and plays a pivotal role in monitoring skeletal burden in metastatic bone disease. In the realm of artificial intelligence, CT data has become a vital component of multimodal diagnostic models. AI-driven radiomics applied to CT images can extract high-dimensional features related to tumor heterogeneity and density, aiding in the differentiation of benign from malignant lesions and predicting histological subtypes (39, 47). Additionally, deep learning algorithms have been successfully employed for automated lesion segmentation and for fusing anatomical CT data with metabolic PET information, thereby enhancing diagnostic accuracy and reducing inter-observer variability in complex cases (40, 41).

**Figure 1:**
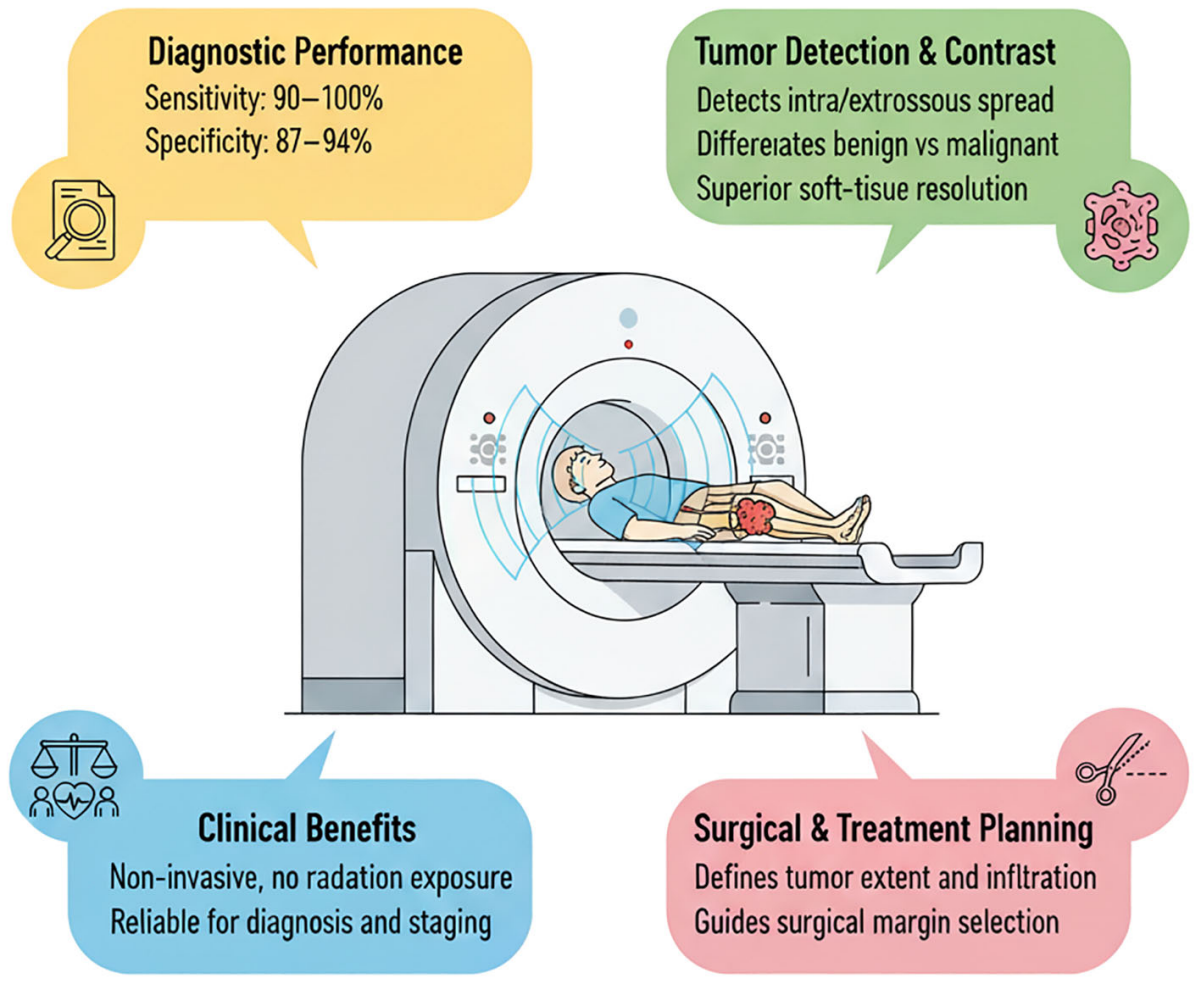
Diagnostic value of MRI in bone tumor evaluation.

Magnetic Resonance Imaging (MRI) serves as the cornerstone of local staging for primary bone tumors, extending far beyond its role as the gold standard for assessing bone marrow infiltration and soft tissue invasion (25). (**Figure 1**). MRI’s diagnostic power is now augmented by AI-driven radiomics. These models extract high-dimensional features, such as texture and entropy, to differentiate complex entities like enchondroma from chondrosarcoma at a sub-visual level (10). Notably, AI models using CNNs have demonstrated capabilities in not only detecting and segmenting tumors but also in differentiating between specific tumor types, such as enchondroma and chondrosarcoma, using these radiomic features (1, 26).

Magnetic resonance imaging (MRI) provides high diagnostic accuracy for bone tumors, with reported sensitivity of 90–100%, specificity of 87–94%, and diagnostic accuracy of 90–96%. Superior soft-tissue contrast enables precise detection of intraosseous and extraosseous tumor spread and differentiation of benign from malignant lesions. MRI is particularly valuable for preoperative planning by delineating tumor extent and infiltration, thereby guiding surgical margin selection and assessing treatment response. As a non-invasive modality with no radiation exposure, MRI represents a reliable tool for diagnosis, staging, and follow-up in bone tumor management.

**Figure 2:**
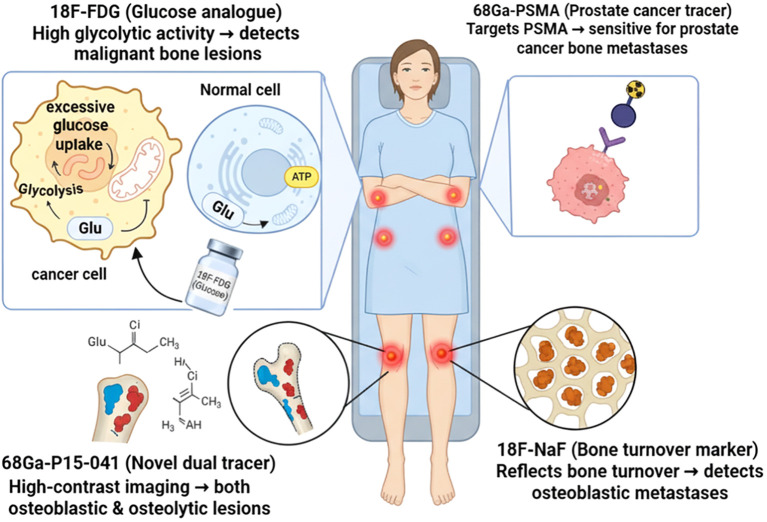
PET tracers for bone tumor imaging and their biological basis.

^18F-FDG (fluorodeoxyglucose) exploits the Warburg effect, detecting malignant lesions through increased glycolysis and excessive glucose uptake in cancer cells. ^68Ga-PSMA targets prostate-specific membrane antigen and provides high sensitivity for prostate cancer bone metastases. ^18F-NaF is a bone turnover marker that accumulates in osteoblastic lesions, reflecting active bone remodeling. The novel dual tracer ^68Ga-P15–041 enables high-contrast imaging of both osteoblastic and osteolytic metastases. Central schematic highlights tracer uptake patterns in a patient with skeletal involvement.

While MRI provides a detailed anatomical view of the local lesion, PET/CT is a fundamental tool for whole-body staging and assessing a tumor’s metabolic activity. Using F-18-FDG as a radiotracer, PET/CT can detect metastases, particularly small pulmonary and lymph node metastases, with superior sensitivity compared to CT alone (27) (**Figure 2**). It is also valuable for evaluating therapeutic response, as a decrease in metabolic activity (measured by SUVmax) correlates with a positive response to chemotherapy (28). AI is now being used to automate and improve the analysis of PET/CT scans by automatically detecting and segmenting lesions, quantifying whole-body skeletal tumor burden, and enhancing image quality through denoising. Studies have shown that AI models offer substantial time savings and higher accuracy and sensitivity for lesion detection than traditional, threshold-based methods (29).

Histopathology remains the definitive gold standard for bone tumor diagnosis and grading. The histological grade is, in fact, the most potent prognostic factor for tumors like chondrosarcoma (25). However, this process is labor-intensive and challenging, with a high degree of inter-observer variability that can lead to diagnostic discrepancies and even changes in patient management. AI is revolutionizing this domain through computational pathology, where models analyze whole-slide images to automatically detect and classify tumors (1, 2, 6). A particularly significant advancement is a new AI model developed at Kyushu University that estimates the viable tumor cell density after chemotherapy. This metric was found to be a more reliable predictor of prognosis than the conventional necrosis rate, which is a key finding that demonstrates the power of AI to refine existing diagnostic paradigms (30).

**Figure 3:**
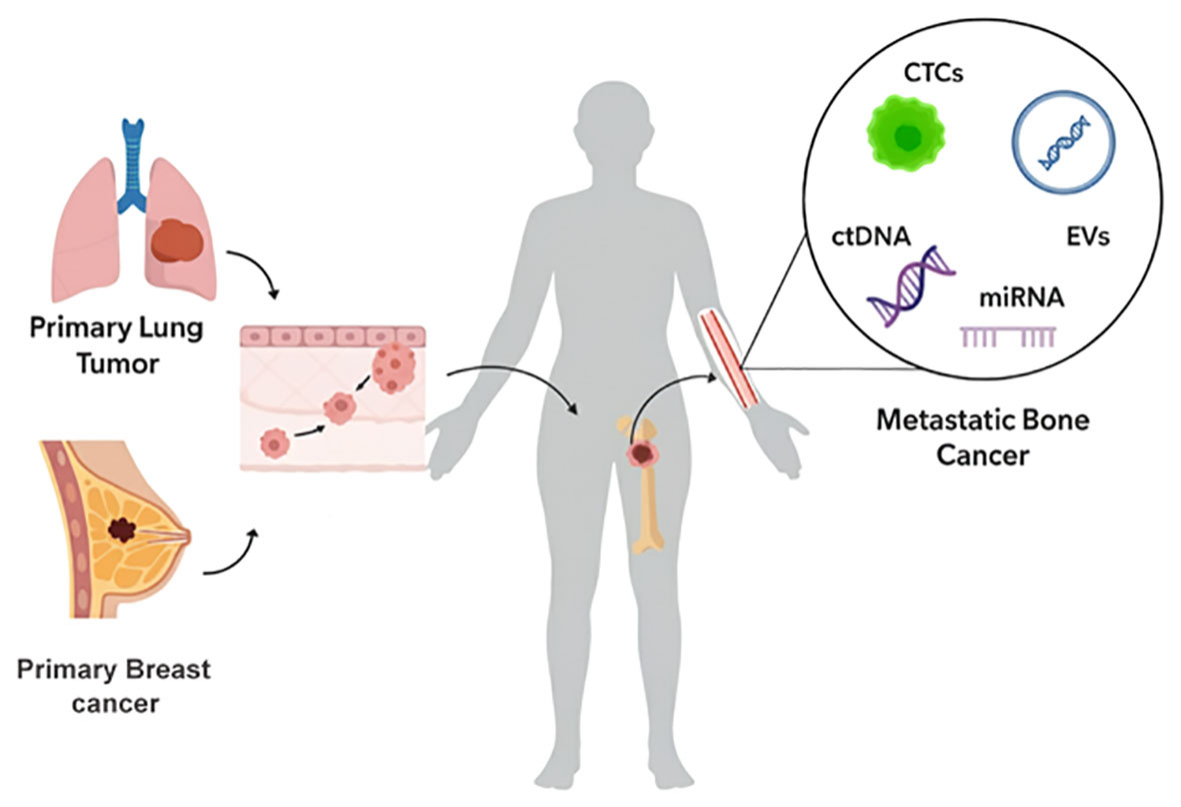
Schematic representation of liquid biopsy-based detection of tumor−derived components in the bloodstream. The illustration depicts circulating biomarkers such as circulating tumor cells (CTCs), circulating tumor DNA (ctDNA), extracellular vesicles (EVs), and microRNAs (miRNA). These biomarkers may originate from primary bone malignancies or from primary tumors in other organs (e.g., lung or breast) that subsequently metastasize to bone. The figure is intended to illustrate the general biological principles of liquid biopsy and does not imply equivalent diagnostic performance across tumor types. While liquid biopsy is well−established for monitoring metastatic bone disease, its application in primary bone tumors remains investigational due to limited and evolving evidence. Accordingly, the figure highlights conceptual pathways rather than validated diagnostic workflows in primary bone oncology.

Liquid biopsy is a minimally invasive technique that detects circulating tumor−derived components such as ctDNA, CTCs, and extracellular vesicles (31) (**Figure 3**). While it can provide a dynamic snapshot of tumor−associated molecular alterations, its clinical utility in primary bone tumors remains limited. Current evidence is preliminary, and challenges such as low ctDNA shedding, tumor heterogeneity, and inconsistent detection rates restrict its diagnostic reliability. Nevertheless, the complex, high−dimensional nature of liquid biopsy–derived multi−omics data makes it suitable for exploratory AI−based analyses. Machine learning models can be applied to genomics, epigenomics, and proteomics datasets to identify non−intuitive molecular patterns and potential biomarker signatures, although these applications remain investigational in the context of primary bone malignancies (32, 33).

## Synthesizing complexity: AI fusion models for integrated diagnosis

The synthesis of data from disparate sources into a single, cohesive diagnostic conclusion represents the pinnacle of multimodal AI. These integrated models move beyond the capabilities of any single modality and begin to operate in a manner that more closely mimics a senior clinician’s thought process, albeit with a speed and consistency that is unattainable for humans. A prime example of an integrated model is the stacked ensemble approach. In one study, Efficient Net deep learning architectures, pre-trained on the ImageNet database, were used to extract features from T1W and T2W MRI studies. These models were then combined in a voting ensemble with a logistic regression model trained on clinical data, specifically patient age, sex, and lesion location. This fusion model successfully discerned between benign and malignant bone lesions with an accuracy of 0.76 and a sensitivity of 0.79, which was considered equivalent to or better than a committee of expert musculoskeletal radiologists (19).

A more advanced approach designed for practical clinical application is the PBTC-TransNet fusion model. Acknowledging that real-world clinical data is often incomplete, this model was specifically designed to classify primary bone tumors as benign, intermediate, or malignant using a combination of X-ray, CT, and MRI data, even when some modalities are missing. The model achieved a satisfactory micro-average AUC of 0.847 on an internal test set and 0.782 on an external test set, demonstrating its strong clinical practicality. The model’s ability to “mirror real-life clinical scenarios” where imaging is incomplete is a crucial step toward its successful clinical deployment (21).

While the full tri-modal integration of imaging, AI, and omics data remains largely absent in the specific field of bone tumors, the concept of radiogenomics fusing imaging features with molecular signatures is a major focus in general oncology and represents a significant future direction (34). AI platforms are being developed to interpret user instructions in natural language to analyze and integrate clinical and genomic data, demonstrating a viable pathway forward for this kind of complex fusion (35).

A review of the performance of integrated AI models indicates a quantitative shift in diagnostic practice. Meta-analyses show that AI algorithms for bone tumor detection have a pooled sensitivity of 84% and specificity of 91%, which is comparable to or even better than clinicians’ performance of 85% and 94%, respectively. Moreover, the integration of AI models has a powerful augmentative effect on human expertise. Clinicians who used AI assistance showed enhanced contouring accuracy and a reduced workload, particularly benefiting less-experienced clinicians (36). This shift from a subjective, qualitative diagnosis to an objective, quantitative, and reproducible one is a foundational change. The superiority of the AI model for estimating viable tumor density is a direct consequence of this shift from a qualitative assessment (necrosis area) to a quantitative one (cell count) (30). This quantitative shift empowers clinicians to better predict treatment response and prognosis, directly advancing the field of precision medicine. The evolution of these diagnostic models, particularly the stacked ensemble architectures (19, 37, 38) and the PBTC-TransNet framework (21), signifies a shift toward ‘diagnostic robustness’ in clinical oncology. By integrating clinical metadata with multimodal imaging, these systems move beyond simple pattern recognition to perform ‘contextual weighting,’ much like a multidisciplinary tumor board. The capacity of these models to maintain high diagnostic accuracy despite missing data or complex clinical presentations demonstrates their practical readiness. This shift ensures that AI is not merely an auxiliary tool for image enhancement but a core component of the diagnostic process, providing objective and reproducible differentiation between benign and malignant lesions. This transition, driven by AI, has transformed data integration from a qualitative clinical task into a high-precision quantitative analysis. This shift has led to measurable increases in diagnostic accuracy, specifically aiming to reduce false-negative rates for earlier detection and minimize false-positives to prevent unnecessary invasive biopsies. Beyond performance, these models serve to lower long-term costs and broaden accessibility by providing expert-level diagnostic support across varied clinical settings. A summary of the specific performance metrics, including sensitivity and specificity for various AI architectures, is detailed in **Table 2**.

**Table 2:** AI and advanced imaging in bone and soft tissue tumors.

Primary focus/objective	Key methodology	Main findings/outcomes	Imaging modality/data type	Performance/key metric	References
AI/ML advances in bone tumor imaging	Review of radiomics, segmentation, and workflow tools	Highlights DL for detection and decision support	MRI, CT, PET/CT	RF AUC: 0.926	(39)
Navigated vs. CT-guided biopsy comparison	Retrospective study (22 navigated vs. 57 CT-guided)	Navigated biopsy showed 100% diagnostic yield	PET, MRI, CT (Fused)	Yield: 100% (Nav) vs. 84% (CT)	(40)
Diagnose bone metastases	Compared SUVmax (PET/CT) with WB-DWI	Combined PET/CT and MR-DWI showed superior efficacy	18F-FDG PET/CT, WB-MRI DWI	Accuracy (P<0.05)	(41)
Primary bone tumor classification	Multimodal DL (Clinical metadata + X-ray)	Multimodal models outperformed image-only models	X-ray, Clinical Metadata	Improved Accuracy	(42)
Boundary segmentation improvement	Multi-modal image fusion network	Fusion improved accuracy over single-modal approaches	Multi-modal (General)	~6.8% accuracy gain	(43)
Automated bone cancer detection	CNN and Karhunen-Loeve feature extraction	High accuracy in differentiating healthy vs. cancerous bone	Histopathology	Accuracy: 97.3%	(44)
Detection of bone metastases	Compared MRI, AC PET, and fused images (765 patients)	Fused Contrast-Enhanced T1W-PET had highest performance	WB-PET/MRI	Sens: 100%, Spec: 92%	(45)
Review of ML/DL (2019-2024)	Systematic review (PRISMA) of RF, CNNs, AlexNet	Advanced models outperformed conventional methods	Clinical/Imaging Data	Accuracy: up to 98.9%	(46)
Benign vs. malignant sacral tumors	Fusion of T2WI/NCCT with DL radiomic nomogram	DLRN model achieved best classification performance	Axial T2WI MRI, NCCT	AUC: 0.961, Acc: 0.889	(47)

AI, Artificial Intelligence; AUC, Area Under the Curve; CNN, Convolutional Neural Network; CT, Computed Tomography; DL, Deep Learning; DLRN, Deep Learning Radiomic Nomogram; DWI, Diffusion-Weighted Imaging; FDG, Fluorodeoxyglucose; ML, Machine Learning; MRI, Magnetic Resonance Imaging; NCCT, Non-Contrast Computed Tomography; PET, Positron Emission Tomography; RF, Random Forest; ROC, Receiver Operating Characteristic; SUVmax, Maximum Standardized Uptake Value; WB, Whole-Body.

## Translating innovation to practice: critical challenges and pathways forward

Despite the promising research outcomes, the path to widespread clinical adoption of multimodal AI in orthopedic oncology is fraught with significant hurdles. The primary challenge is not a lack of technical capability, but a deep-seated issue of trust and the logistical complexity of integrating AI into established healthcare workflows. A major foundational hurdle is the scarcity and heterogeneity of data. Primary bone tumors are rare, which limits the availability of the large, high-quality, and well-annotated datasets that are essential for training robust deep learning models. This is compounded by the lack of standardized imaging protocols, which complicates the reproducibility and generalizability of models across different institutions (1). Furthermore, data from disparate sources such as EHRs, imaging archives, and laboratory information systems often lacks consistency and requires significant effort to be structured and standardized in a manner that supports scalable AI development (48). Beyond technical hurdles, the ‘black box’ nature of deep learning remains a primary barrier to clinical integration. This lack of transparency can obscure the reasoning behind AI-generated insights, making it difficult for clinicians to validate results against their expert judgment (49–52).

**Figure 4:**
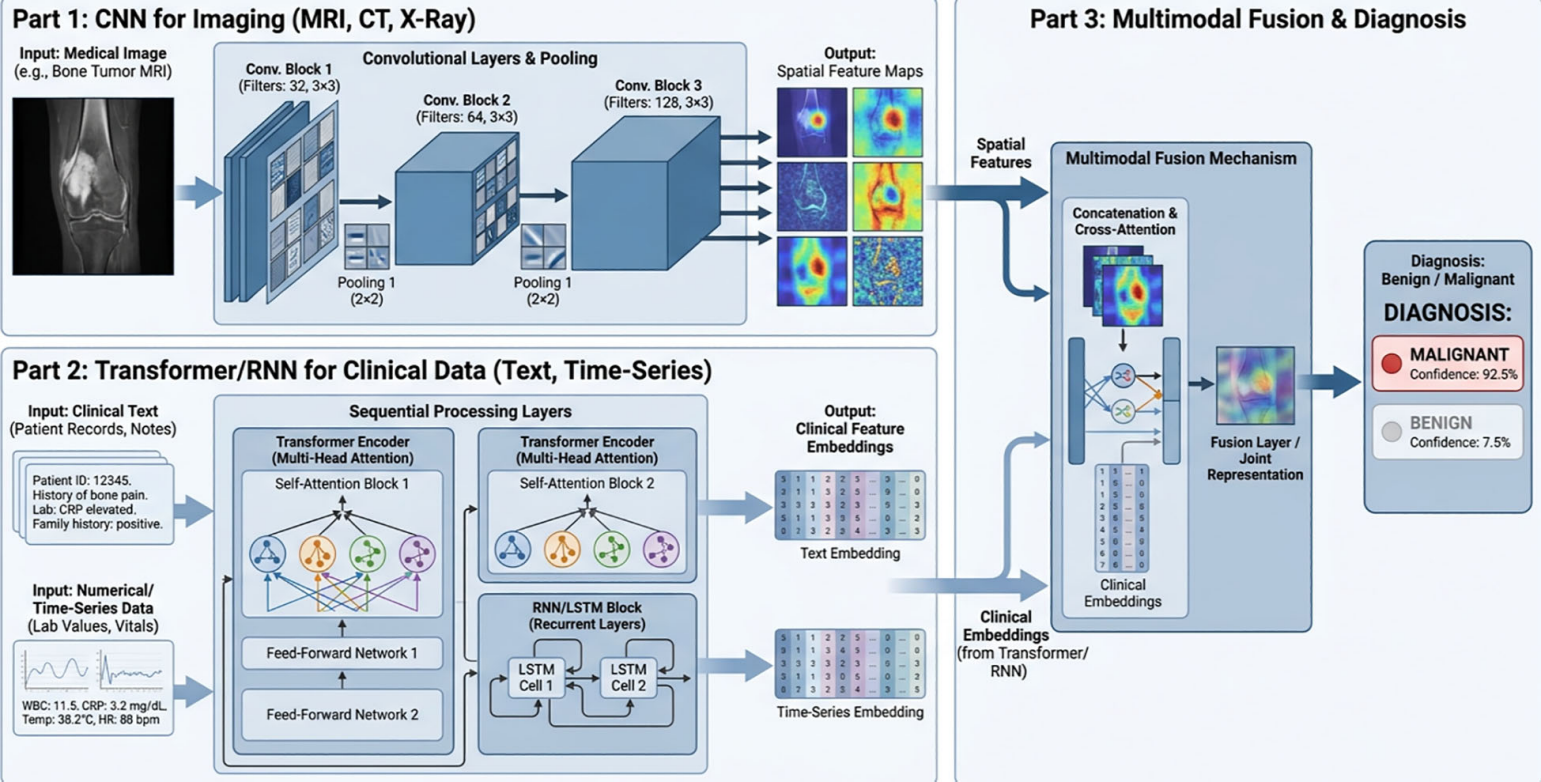
Schematic overview of deep learning architectures in multimodal bone tumor diagnosis. This diagram illustrates the workflow of key deep learning models. Convolutional Neural Networks (CNNs) process imaging data (e.g., MRI, CT) to extract spatial features such as tumor boundaries and texture. Recurrent Neural Networks (RNNs) and Transformers handle sequential or structured data (e.g., clinical history, genomic sequences) to capture contextual relationships. In Multimodal Fusion, the features extracted from these distinct pathways are integrated to produce a unified diagnostic prediction, enhancing accuracy beyond what any single modality could achieve alone.

Therefore, transitioning toward Explainable AI (XAI) is essential. Rather than acting as an autonomous decision-maker, XAI serves as a transparent decision-support tool that provides interpretable rationales. This ensures that the physician remains the central authority, using AI-driven data to augment, rather than replace, clinical expertise. By reframing the ‘black box’ as a socio-technical challenge, we emphasize that future AI adoption depends on building a collaborative framework that aligns with the principles of evidence-based medicine and enhances the patient-physician relationship (53–55) (**Figure 4**).

## Future directions and conclusion

The synthesis of current research underlines a significant advancement in bone tumor diagnostics through the adoption of multimodal AI. Our analysis demonstrates that by integrating disparate data streams radiological, histopathological, and genomic AI models achieve a level of diagnostic precision that consistently surpasses unimodal approaches. Interpreting these results, it becomes clear that the clinical value of such systems lies in their ability to address the inherent biological heterogeneity of bone tumors, which a single modality cannot fully capture. This structured integration does not merely improve accuracy; it provides a more robust, evidence-based framework for multidisciplinary decision-making, effectively bridging the gap between high-dimensional data and clinical utility. The future of multimodal AI in orthopedic oncology will be defined by a strategic evolution from a static diagnostic tool to a dynamic, prognostic, and personalized guidance system. The next generation of models will move beyond simply classifying a tumor to predicting a patient’s response to chemotherapy, the risk of metastasis, and long-term prognosis. This will require the full-scale integration of a patient’s entire clinical data stream, including longitudinal imaging and molecular data from liquid biopsies, to create a real-time, comprehensive digital twin of their disease.

A critical frontier is the full-scale integration of imaging and omics data, a field known as radiogenomics. This fusion is particularly vital in bone oncology, where low levels of circulating tumor DNA (ctDNA) often limit the efficacy of liquid biopsies in diseases like osteosarcoma. By leveraging AI to correlate visual radiomic patterns with underlying molecular changes, these models can compensate for biological marker scarcity, providing a ‘virtual biopsy’ that enhances the reliability of differential diagnosis. This approach allows for a more precise distinction between histologically similar entities, such as osteosarcoma and Ewing sarcoma, and significantly improves the specificity of malignant-vs-benign classification, moving beyond the limitations of traditional single-biomarker strategies. AI platforms are already being developed to perform these kinds of complex analyses by interpreting natural language instructions, demonstrating a path forward for this integrated approach.

To overcome the foundational hurdles of data scarcity and privacy, future research must prioritize multi-center collaborations and the use of technical solutions like federated learning. This approach will allow AI models to be trained on decentralized data without compromising patient privacy or requiring the movement of sensitive information. Simultaneously, research must focus on developing models that are not only accurate but also transparent and explainable. Explainable AI is essential for building clinician trust and ensuring patient safety, as it provides a pathway for physicians to understand and validate the AI’s recommendations. Crucially, by highlighting the diagnostic reasoning, these transparent models serve as pedagogical tools for trainees, ensuring they remain active decision-makers rather than passive observers. This human-in-the-loop approach preserves clinical expertise even as the specialist workforce evolves, ensuring AI augments rather than replaces the development of new experts. Furthermore, addressing current data gaps specifically the lack of longitudinal post-treatment outcomes and standardizing image resolution across protocols will be vital, as inconsistent data quality remains a primary barrier to model generalizability.

In conclusion, multimodal AI holds the potential to transform orthopedic oncology by creating a unified, data-driven diagnostic and prognostic framework. By systematically integrating and analyzing heterogeneous data streams, these systems can empower clinicians with unprecedented insights, enable more precise and personalized treatment strategies, and ultimately improve patient outcomes in a field that has seen limited advancements for decades. The future of bone tumor diagnosis will be defined by the success of these integrated, intelligent, and collaborative systems that can bridge the gap between technical innovation and trusted clinical practice.
